# Some microeconometric evidence on the relationship between health and income

**DOI:** 10.1186/s13561-017-0163-5

**Published:** 2017-08-14

**Authors:** Amélie Adeline, Eric Delattre

**Affiliations:** THEMA, University of Cergy-Pontoise, 33 Bd. du Port, Cergy, 95000 France

**Keywords:** IOO, I14, D31, Health inequalities, Income inequalities, Self-reported health, Europe

## Abstract

This paper examines the association between income, income inequalities and health inequalities in Europe. The contribution of this paper is to study different hypotheses linking self-perceived health status and income, allowing for the identification of different mechanisms in income-related health inequalities. Using data from the Survey of Health, Ageing and Retirement in Europe (15 countries), we take the advantage of the cross-sectional and longitudinal nature of this rich database to make robust results. The analyses (coefficient estimates as well as average marginal effects) strongly support two hypotheses by showing that (i) income has a positive and concave effect on health (Absolute Income Hypothesis); (ii) income inequalities in a country affect all members in a society (strong version of the Income Inequality Hypothesis). However, our study suggests that, when considering the position of the individual in the income distribution, as well as the interaction between income inequalities and these rankings, one cannot identify individuals the most affected by income inequalities (which should be the least well-off in a society according to the weak version of the Income Inequality Hypothesis). Finally, the robustness of this study is emphasized when implementing a generalized ordered probit to consider the subjective nature of the self-perceived health status to avoid the traps encountered in previous studies.

## Background

The last few years have seen unprecedented attention to an attempt by policy makers, policy advisers and international institutions to reduce health inequalities. To do so, they usually focus on the access to healthcare, given that such policies allow to improve the health of lower income groups [28, 34]. Improving equality of access to healthcare is however not the sole public policy which can favor health equality. In particular, it has been widely said that income and income inequalities are associated to health status; thus, any public policy which influences income and/or income inequalities might influence health. In this way, studying the relationship between income, income inequalities and health is interesting per se. With these elements in mind, this paper confronts on an empirical basis three hypotheses. The first one, called the Absolute Income Hypothesis, was initially introduced by Preston [29] and states that there is a positive and concave relationship between income and health.^1^ Higher incomes can provide means for purchasing a better health status. The second one is the strong version of the Income Inequality Hypothesis and it asserts that the health status is determined by income inequalities within a society. Thus, the health of all individuals is affected by an increase or a decrease in income inequalities. The last one, a weak version of the Income Inequality Hypothesis, says that income inequalities are a threat to individuals placed at the lower end of the income distribution. This last hypothesis implies that income inequalities do not impact low income people and high income people in the same magnitude.

Various authors have studied the Absolute Income Hypothesis mainly in the United States, using different health measures, like self-perceived measures [26], life expectancy [10] and other health outcomes [8, 12]. Fiscella and Franks [13], Kennedy et al. [20], Van Doorslaer et al. [32], Wagstaff et al. [33] focus on the strong version of the Income Inequality Hypothesis and show that income inequalities in a society also matter in order to explain the average health status measured by self-perceived measures (mostly in the United States). Concerning the weak version of the Income Inequality Hypothesis, there are few empirical studies which investigate it, with the exception of Mellor and Milyo [27] in the United States, Li and Zhu [21] in China or Hildebrand and Van Kerm [15] in Europe. Importantly, the strong version of Income Inequality Hypothesis and the weak version of Income Inequality Hypothesis are non-nested given that the weak version considers the rank of individuals and an interaction term between the rank and the income inequalities index whereas the strong version does not. Thus, both versions can be valid when income inequalities in a society are negatively associated to the health of all individuals, and more particularly the health of people ranked at the lower end of the income distribution. However, the authors previously mentioned focus mainly on one of the versions in the best case (mainly on data from the United States), without comparing them. This paper aims at filling these gaps by looking at the three hypotheses, using the same European data, in order to give more insight about efficient public policies which should be implemented in Europe. Finally, studying these three hypotheses at the same time allows to highlight different mechanisms between health and income.

In this paper, we test the three above hypotheses with the Survey of Health, Ageing, and Retirement in Europe (SHARE), using mainly the fifth wave of this survey (2015 release), as well as the pooled version of the survey in robustness. We use self-perceived health status as our health outcome. This type of subjective measure is sometimes criticized but it is similar to the ones used by Mackenbach et al. [26], Fiscella and Franks [13] and Hildebrand and Van Kerm [15]. Furthermore, some authors show that these subjective measures are not biased [1]. Lastly, even if this type of measure can be criticized because of interpersonal comparison issues, authors prove that some econometric models tackle these problems [22] (see “Robustness checks” subsection for some robustness checks in which we explicitly consider this issue).

The paper is organized as follows. “Literature review: the relationship between income inequalities and health” section presents formally the three hypotheses that we will test empirically. “Method” section describes the SHARE dataset as well as the baseline econometric specification. In “Results” section we present the results and some robustness checks. “Conclusion” section concludes the paper.

## Literature review: the relationship between income inequalities and health

Inequalities in health refer to the close relationship between health and membership in a group characterized by incomes, where income is an individual social determinant. This section formally presents the three hypotheses mentioned in the introduction, as well as some related literature. We should mention that, in this literature review, we transcribe terminology employed by authors which reflects causal relationships even if cross-sectional databases are used or some endogeneity might be at play.

### The Absolute Income Hypothesis

From an early stage in the debate, the Absolute Income Hypothesis states that the relationship between health and income is positive and concave [29], meaning that people with higher incomes have better health outcomes, but income inequalities have no direct effect on health. As a result, the concavity of the relationship between individual income and health status is a necessary condition to assess the efficiency of redistributive policies, in which transferring a given amount of money from rich people to poor people will result in an improvement of the average health.

The individual-level relation between income and health is specified as follows:

1 $$  h_{i} = \beta_{0} + x_{i}\beta_{1} + x_{i}^{2}\beta_{2}+Z_{i}\gamma + \epsilon_{i}  $$

where *h*_*i*_ represents the health status of individual *i* (objective or subjective measures); *x*_*i*_ is the income of individual *i*; *Z*_*i*_ is a set of individual specific control variables^2^; and *ε*_*i*_ is the error term coming from differences in individual health. The concavity effect is legitimized if *β*_1_ is positive, *β*_2_ is negative, and $\frac {\partial h_{i}}{\partial x_{i}}>0$.

A strong link between health and income has been demonstrated in a large number of empirical studies, and a concave relationship between the two is found. Preston [29] explains that the impact of additional income on mortality is greater among the poor than richer people. Ettner [12], using three US surveys, finds that increases in income improve mental and physical health but also increase alcohol consumption. Then, Mackenbach et al. [26] show that a higher income is associated with better self-assessed health in Europe. Using mortality rates, Cutler et al. [10] conclude the same thing in the United States. Theodossiou and Zangelidis [31], using data on individuals aged between 50 and 65 from six European countries, find a positive but small effect of income on health. More recently, Carrieri and Jones [8] analyze the effect of income on blood-based biomarkers and find a positive and concave effect of income on health.

### The strong version of Income Inequality Hypothesis

Some researchers affirm that income inequalities in a society are equally important in determining individual health status. The key difference between the Absolute Income Hypothesis and the strong version of Income Inequality Hypothesis stems from the fact that the latter explicitly considers the effect of income inequalities on health while the former only takes into account the concavity assumption between health and income. Mellor and Milyo [27] specifically define two versions of this hypothesis: the strong version and the weak version. The strong version of the Income Inequality Hypothesis implies that, whatever the level of income, the health of all individuals in a society is equivalently affected by income inequalities in this society. In this way, both the well-off and poor people are impacted by income inequalities. These may be a public bad for all members in a society since income inequalities are a threat to the health of all individuals. We can thus identify an individual effect (a micro part) which is assimilated to the Absolute Income Hypothesis and an aggregate effect (a macro part) which corresponds to the relationship between individual health and income inequalities in a society. Theoretically, the strong version of the Income Inequality Hypothesis is specified as follows:

2 $$ h_{ij} = \beta_{0} + x_{i}\beta_{1} + x_{i}^{2}\beta_{2} + \delta II_{j}+Z_{i}\gamma + \epsilon_{ij}  $$

which is an expansion of Eq. (1) with the introduction of *II*_*j*_ as a measure of income inequalities in a society *j* (corresponding to the macro part explained above); where *h*_*ij*_ represents the health status of individual *i* in a society *j*.

This hypothesis has been empirically tested mainly on data from developed countries (principally in the United States). Tests have been conducted at both the individual level and the aggregate level. At the aggregate level, a number of studies try to demonstrate an association between income inequalities and public health and the results are contrasted [17, 25, 30]. At the individual level, Kawachi et al. [19], Kennedy et al. [20], and Fiscella and Franks [13] all find a negative association between income inequalities and self-perceived health. However, Van Doorslaer et al. [32] find no effect of income inequalities on an objective health measure, the McMaster health utility index, derived from the self-perceived health status. Finally, other authors test the impact of income inequalities on malnutrition [33] or health service use [23] and find contrasted results.

The strong version focuses on the direct ties between health and income inequalities. There are several potential pathways through which income inequalities might be negatively related to an individual’s health. Kawachi and Kennedy [18] summarize three plausible mechanisms linking income inequalities to health. The first one is that disinvestment in human capital is linked to income inequalities. In states with high income inequalities, educational outcomes are negatively impacted when a smaller proportion of the state budget is spent on education which creates differences in education and thus in income. High income disparities may translate into lower social spending because interests of richer persons begin to diverge from other people in societies where inequalities rise. Thus, reducing social spending turns into a decrease in life opportunities for poorer people and thus an increase in inequalities (see also [14]). The second mechanism is that income inequalities lead to the erosion of the “features of social organization that facilitate cooperation for mutual benefit”. In other words, Kawachi and Kennedy [18] interpret this mechanism as the erosion of the “social capital”, corresponding to the set of collective resources an individual can put together. This may be the access to public services, the feeling of security, the characteristics of the relatives or the community solidarity (Grignon et al.: Mesurer l’impact des déterminants non médicaux des inégalités sociales de santé, unpublished). Here we focus on the solidarity argument. This one is important for the maintenance of population health. Kawachi and Kennedy [18] made a study using the General Social Survey where each indicator of social capital (like the degree of mistrust or levels of perceived reciprocity) was correlated with lower mortality rates. An increasing level of mistrust between the members of a society was due to the development of the distance between the well-off’s expectation and the ones of poorer people. Unfortunately this result implies a growth of a latent social conflict. As a result, when health is associated to the erosion of social capital, this seems to be towards the transition of social policies which are detrimental to poor people, implying unequal political participation. A lower turnout at elections is perceived among states with low levels of interpersonal trust. These states are less likely to invest in policies that ensure the security of poorer people in a society. Finally less generous states are likely to provide less hospitable environments for these individuals. The last mechanism is that income inequalities are correlated to unhealthiness through stressful social comparisons. In this case, a technique in anthropology called “cultural consensus analysis” is used to take into account the psychosocial effects of social comparisons. Indeed, many communities have a common cultural model of the standard of living. This technique involves interviewing people and observing if individuals succeed in achieving the cultural model of lifestyle. This aspect can be seen as the satisfaction individuals have with their life. However, it should be noticed and not forgiven that a possible endogeneity issue can appear with this mechanism connected to the life satisfaction of individuals.

### The weak version of Income Inequality Hypothesis

The second version of the Income Inequality Hypothesis is the weak one. According to this hypothesis, people who are more likely to have poorer health are the ones who feel more economically disadvantaged than their peers in a reference group. As a result, it specifically suggests that only the least well-off are hurt by income inequalities in a society. The damaging effect of these inequalities on health decreases with a person’s income rank. Indeed, for an individual, stress and depression leading to illness may be linked to the fact of having a low relative income when compared to another person [9]. The main concern is thus on the difficulties that an individual may face when he is situated at the bottom of the social ladder. Theoretically, the weak version of the Income Inequality Hypothesis is specified as follows:

3 $$\begin{array}{*{20}l} h_{ij} &= \beta_{0} + x_{i}\beta_{1} + x_{i}^{2}\beta_{2} + \delta II_{j} + \theta R_{ij} \\ & \quad + \eta R_{ij}*II_{j}+Z_{i}\gamma +\epsilon_{ij} \end{array} $$

which is an expansion of Eq. (2) where we introduce *R*_*ij*_ as a person’s rank, and the interaction between inequalities and a person’s rank (*R*_*ij*_∗*II*_*j*_) to allow the effects of income inequalities to vary by the relative income level in a society. The interaction term allows us to know how income inequalities are related to people with lower levels of income, compared to other people. Therefore, this hypothesis suggests that the breadth of the difference between rich people and poor ones accounts for the health. When testing this equation, *δ* underlines the strong version of the Income Inequality Hypothesis whereas *θ* and *η* specifically refer to the weak version. Thus, if the three previous coefficients are significant and have the right signs, then both the strong and the weak version are correct, meaning that everybody’s health is associated to income inequalities, and in particular people who are at the lower end of the income distribution. On the other hand, whether only *δ* (or *θ* and *η* respectively) is significant implies that only the strong version (*resp.* the weak version) is satisfied.

As explained in the introduction, only few researches focus on this hypothesis. Mellor and Milyo [27] use data from the Current Population Survey and find no consistent association between income inequalities and individual health. On the other hand, Li and Zhu [21], using data from China, find that income inequalities are detrimental for people who are at the lower end of the income hierarchy. Finally, Hildebrand and Van Kerm [15] also test the hypothesis that income inequalities may affect only the least well-off in a society using the European Community Household Panel but find no evidence supporting it.

## Method

### The data

#### The survey

The Survey of Health, Ageing and Retirement in Europe (SHARE) is a multidisciplinary and cross-national panel database of micro data on health, socio-economic status and social and family networks of more than 123,000 individuals aged 50 and over from many European countries and Israel [7]. Since 2004, SHARE asks questions throughout Europe to a sample of households with at least one member who is 50 and older. These households are re-interviewed every two years in the panel. SHARE is part of a context of an ageing population. It is the European Commission which has identified the need for scientific knowledge about ageing people in Europe.^3^ In fact, people of the European Innovation Partnership on Active and Health Ageing project estimate that in 2050, one in three Europeans will be over 60 years old and one in ten will be over 85 years old. The SHARE survey was then constructed in the different European countries under the leadership of Professor Axel Börsch-Supan. In addition, SHARE is harmonized with the Health and Retirement Study (in the United States - HRS) and the English Longitudinal Study of Ageing (UK - ELSA).

The first wave (2004-2005, 27,014 individuals) and the second one (2006-2007, 34,393 individuals) were used to collect data on health status, medical consumption, socio-economic status and living conditions. The 2008-2009 survey (Wave 3 - “SHARELIFE”) was extended to life stories by collecting information on the history of the respondents. The number of participants increased from 12 countries in wave 1, to 15 (+ Ireland, Israel, Poland and Czech Republic) in wave 2, and the third wave contains information about 14 countries. The fourth wave (2010-2011), is a return to the initial questionnaire of the first two waves. It collects data from 56,675 individuals in 16 European countries. Finally, the fieldwork of the fifth wave of this survey was completed in 2013. The following countries are included in the scientific release of 2015: Austria, Belgium, Switzerland, Czech Republic, Germany, Denmark, Estonia, Spain, France, Israel, Italy, Luxembourg, Netherlands, Sweden, and Slovenia. This wave contains the responses of 63,626 individuals. We focus on the fifth wave [3] in order to have a great number of individuals who come from different countries. Moreover, in order to test and compare the three hypotheses linking health and income, one has to use the same set of observations (e.g. the fifth wave of the SHARE survey). We do not make our analysis using directly the pooled database since all the control explanatory variables are not available in each waves, which is a limitation of this database. Moreover, we also focus on the pooled database (waves 1, 2, 4 [4–6] and 5) in order to make our results more robust (the third wave is not considered in the pooled database since it does not contain the same information as the other ones).

The advantage of the SHARE database is that it has many individual variables on health, socioeconomic status and income to perform this research. However, researchers should be also aware of the potential disadvantage of this database. Indeed, Börsch-Supan et al. [7] explain that in some waves there are a relative low response rates and moderate levels of attrition (even though the overall response rate is high compared to other European and US surveys^4^) which are due to the economic crisis faced by some countries, implying a decrease in the participation rates. Due to this attrition, we thus focus on the fifth wave of this survey instead of the pooled database. Nonetheless, we present the results using the pooled database as a robustness test.

#### Indexes for the measurement of income inequalities

In this study, we want to underline the effects of income inequalities on health and this is why we need a measurement of income inequalities. The Gini coefficient, as well as the Theil index are two well-known indexes which can be used.

Algebraically, the Gini coefficient is defined as half of the arithmetic average of the absolute differences between all pairs of incomes in a population, and then the total is normalized on mean income. If incomes in a population are distributed completely equally, the Gini value is zero, and if one person has all the incomes in a society, the Gini is one. The Gini coefficient can be illustrated through the Lorenz curve. However, the Gini coefficient does not take into account the income distribution since different Lorenz curves may correspond to the same Gini index.^5^ In other words, it does not distinguish between inequalities in low income group and high income ones. Formally, the Gini coefficient is:

4 $$ Gini = \frac{2\sum_{i}iy_{i}}{N\sum y_{i}}-\left(\frac{N+1}{N}\right)  $$

with *y*_*i*_ representing the income of the population sorted and ranked, from the lowest decile group to the top decile group, and *N* representing the total population.

As a result, one of the solution is to use the Theil index which measures income inequalities. The Theil index is:

5 $$ Theil = \frac{1}{N} \sum_{i}\frac{y_{i}}{\bar{y}} \ln \left(\frac{y_{i}}{\bar{y}}\right)  $$

where $\bar {y}$ is the mean income per person (or expenditure per capita). In order to normalize the Theil index to vary between zero and one, we divide it by *ln*(*N*).^6^ It measures a “distance” of the real population and the “ideal” egalitarian state where everyone have the same income.

Since the Gini coefficient does not take into account the income distribution, most of the following tables of results will be displayed using the Theil index.

#### Descriptive statistics - an overview

**Fig. 1:**
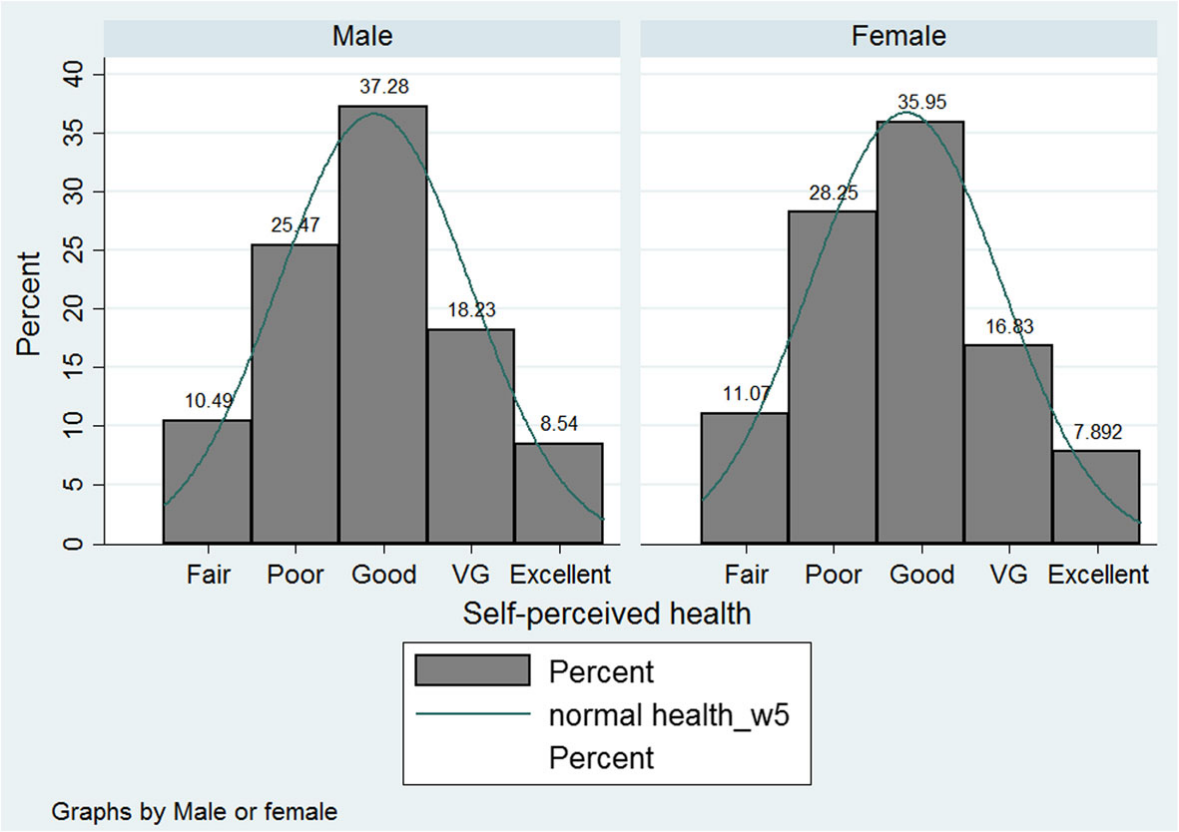
Self-perceived health in Europe

**Fig. 2:**
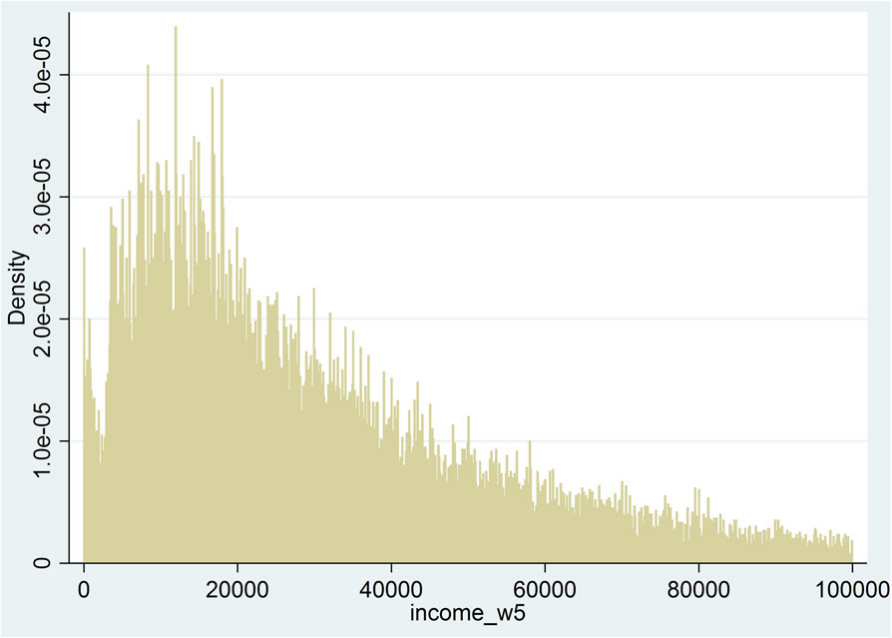
Distribution of income in Europe

**Fig. 3:**
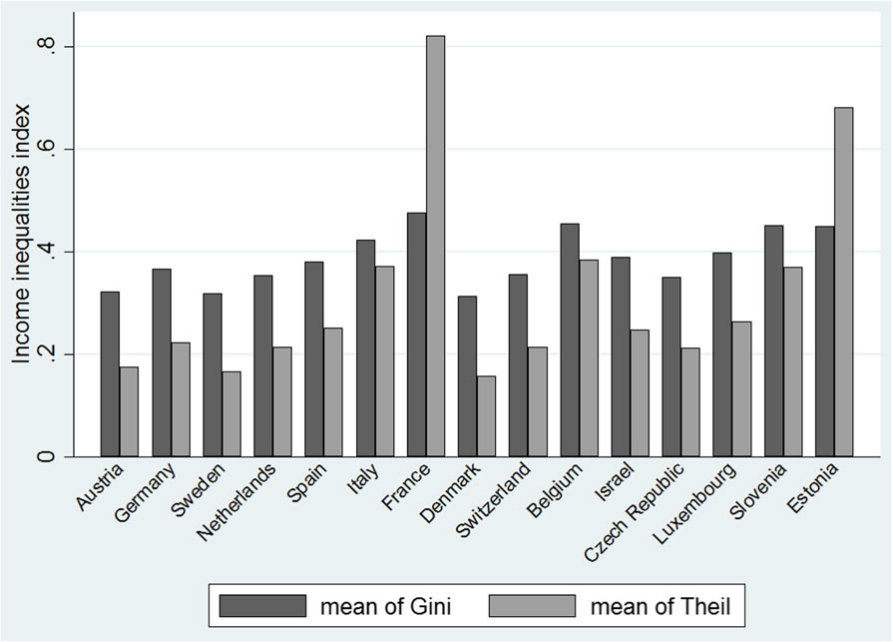
Income inequalities indexes in Europe

In this paper, the data used are from the fifth wave of the SHARE survey. This wave includes responses from 63,626 respondents aged 50 and over, living in 15 different countries. Thus, this survey aims to provide information on health, income, activities and other features of the elderly. In one hand, the variable of interest is the health which is defined in the database as the self-perceived health status. Individuals are asked to classify their health using ordered qualitative labels from “poor” to “excellent. The Fig. 1 characterizes the distribution of the health variable among individuals aged 50 and older by gender for all countries. As we can see the majority of inhabitants reports being in a good health. In the other hand, one of our main determinant of health is the income. This variable can be seen as a proxy for well-being, that is to say a factor which allows individuals to improve the living standards. In the database, it corresponds to the sum of individual imputed income for all household components. Figure 2 shows the distribution of income of people aged 50 and over in the fifth wave where the mean is about 36,000€. Moreover, the income inequality hypothesis includes an indicator for the measurement of income inequalities (see Fig. 3). In this paper, we use either the Gini index or the Theil index. The mean of the Gini index in Europe is 0.39 which corresponds to a rather egalitarian society. The mean of the Theil index in Europe is 0.33 which is also rather egalitarian. In our analysis we include others variables such as the age, the marital status, the education, the job situation, dummies for the countries and the gender, and the GDP of the countries (see Tables 2, 3, 4 and 5 in the Appendix for further information). Finally, the pooled data (waves 1, 2, 4 and 5) contains 181,708 observations, where each individual is present on average 2.9 years in the panel.

### The ordered probit model

To model the association between self-perceived health and other socioeconomic status and test the hypotheses, we use an ordered probit specification. When the self-perceived health status outcome is denoted as *h*_*i*_, the model can be stated as:

6 $$\begin{array}{*{20}l} h_{i}=j \qquad \text{iff} \quad \mu_{j-1}<h_{i}^{*}\leq\mu_{j}, \\ &\text{for} \quad j=1, 2, 3, 4, 5 \end{array} $$

The latent variable specification of the model that we estimate can be written as:

7 $$ h_{i}^{*}= x_{i}\beta + \epsilon_{i}  $$

where $h_{i}^{*}$ is a latent variable which underlies the self-reported health status^7^; *x*_*i*_ is a set of observed socioeconomic variables; and *ε*_*i*_ is an individual-specific error term, which is assumed to be normally distributed.

In this data, the latent outcome $h_{i}^{*}$ is not observed. Instead, we observe an indicator of the category in which the latent indicator falls. As a result the observed variable is equal to 1, 2, 3, 4 or 5 for “poor”, “fair”, “good”, “very good” or “excellent” with this probability:

8 $$  P(y=j|x)=F(\mu_{j}-x_{i}\beta)-F(\mu_{j-1}-x_{i}\beta)  $$

The interval decision rule is:

1. *h*_*i*_=1 if $h_{i}^{*} \leq \mu _{1}$;
2. *h*_*i*_=2 if $\mu _{1} < h_{i}^{*} \leq \mu _{2}$;
3. *h*_*i*_=3 if $\mu _{2} < h_{i}^{*} \leq \mu _{3}$;
4. *h*_*i*_=4 if $\mu _{3} < h_{i}^{*} \leq \mu _{4}$;
5. *h*_*i*_=5 if $h_{i}^{*} > \mu _{4}$.

In this model, the threshold values (*μ*_1_,*μ*_2_,*μ*_3_,*μ*_4_) are unknown. We do not know the value of the index necessary to shift from very good to excellent. In theory, the threshold values are different for everyone.

## Results

### Economic results and discussion

Table 1 reports coefficient estimates for all estimated ordered probit models when income inequalities are measured using the Theil index.^8^ The fifth wave gives us access to 63,626 observations and we also display results of the pooled database for sake of robustness (see Table 6 in the Appendix section). Results in the first column reports the estimated coefficients for the absolute income hypothesis while results in columns two and three provide tests of both the strong version and the weak version of the income inequality hypothesis.

**Table 1:** Results of the ordered probit regressions for Wave 5

Variables	Absolute income	IIH	
	Hypothesis	Strong version	Weak version
Income	$\underset {(1.22\text {e-}07)}{1.84\text {e-}06^{{ ***}}}$	$\underset {(1.20\text {e-}07)}{1.84\text {e-}06^{{ ***}}}$	$\underset {(1.44\text {e-}07)}{1.89\text {e-}06^{{ ***}}}$
Income squared	$\underset {(1.55\text {e-}14)}{-2.06\text {e-}13^{{ ***}}}$	$\underset {(1.50\text {e-}14)}{-2.04\text {e-}13^{{ ***}}}$	$\underset {(1.73\text {e-}14)}{-2.09\text {e-}13^{{ ***}}}$
*Quintiles of income: Reference - Q5*			
Quintile 1			$\underset {(0.029)}{-0.258^{{ ***}}}$
Quintile 2			$\underset {(0.028)}{-0.201^{{ ***}}}$
Quintile 3			$\underset {(0.027)}{-0.115^{{ ***}}}$
Quintile 4			$\underset {(0.026)}{-0.053^{{ ***}}}$
Index of inequalities (II) - Theil		$\underset {(0.024)}{-0.403^{{ ***}}}$	$\underset {(0.049)}{-0.838^{{ ***}}}$
Interaction quintile 1 and II			$\underset {(0.069)}{0.115^{{ *}}}$
Interaction quintile 2 and II			$\underset {(0.068)}{0.114^{{ *}}}$
Interaction quintile 3 and II			$\underset {(0.068)}{0.023}$
Interaction quintile 4 and II			$\underset {(0.068)}{0.062}$
GDP		$\underset {(4.53\text {e-}07)}{1.99\text {e-}06^{{ ***}}}$	$\underset {(0.049)}{0.0001^{{ ***}}}$
Age	$\underset {(0.006)}{0.037^{{ ***}}}$	$\underset {(0.006)}{0.019^{{ ***}}}$	$\underset {(0.006)}{0.037^{{ ***}}}$
Age squared	$\underset {(0.00004)}{-0.0004^{{ ***}}}$	$\underset {(0.0004)}{-0.0003^{{ ***}}}$	$\underset {(0.00004)}{-0.0004^{{ ***}}}$
Years of education	$\underset {(0.001)}{0.034^{{ ***}}}$	$\underset {(0.001)}{0.028^{{ ***}}}$	$\underset {(0.001)}{0.026^{{ ***}}}$
Gender =1 if women	$\underset {(0.009)}{0.003}$	$\underset {(0.009)}{0.005}$	$\underset {(0.009)}{0.007}$
*Marital Status: Reference - Married*			
Registered partnership	$\underset {(0.035)}{-0.042}$	$\underset {(0.035)}{-0.006}$	$\underset {(0.035)}{0.058^{{ *}}}$
Married, not living with spouse	$\underset {(0.039)}{-0.094^{{ **}}}$	$\underset {(0.039)}{0.004}$	$\underset {(0.039)}{-0.076^{{ **}}}$
Never married	$\underset {(0.019)}{-0.071^{{ ***}}}$	$\underset {(0.019)}{0.023}$	$\underset {(0.019)}{0.023}$
Divorced	$\underset {(0.015)}{-0.045^{{ ***}}}$	$\underset {(0.016)}{0.068^{{ ***}}}$	$\underset {(0.018)}{0.032^{{ **}}}$
Widowed	$\underset {(0.014)}{-0.024^{{ *}}}$	$\underset {(0.015)}{0.055^{{ ***}}}$	$\underset {(0.014)}{0.015}$
*Job Situation: Reference Retired*			
Employed	$\underset {(0.014)}{0.253^{{ ***}}}$	$\underset {(0.014)}{0.224^{{ ***}}}$	$\underset {(0.014)}{0.246^{{ ***}}}$
Unemployed	$\underset {(0.028)}{-0.212^{{ ***}}}$	$\underset {(0.028)}{-0.103^{{ ***}}}$	$\underset {(0.028)}{-0.176^{{ ***}}}$
Permanently sick	$\underset {(0.026)}{-1.25^{{ ***}}}$	$\underset {(0.026)}{-1.069^{{ ***}}}$	$\underset {(0.026)}{-1.207^{{ ***}}}$
Home-maker	$\underset {(0.017)}{-0.059^{{ ***}}}$	$\underset {(0.017)}{-0.064^{{ ***}}}$	$\underset {(0.017)}{-0.056^{{ ***}}}$
Other	$\underset {(0.031)}{-0.236^{{ ***}}}$	$\underset {(0.031)}{-1.169^{{ ***}}}$	$\underset {(0.031)}{-0.207^{{ ***}}}$
*Mechanisms IIHs:*			
1 ^st^: % Health expenditure in GDP		$\underset {(0.003)}{0.077^{{ ***}}}$	
2 ^nd^: Received help from others		$\underset {(0.006)}{-0.179^{{ ***}}}$	
2 ^nd^ bis: Given help from others		$\underset {(0.0001)}{0.001^{{ ***}}}$	
3 ^rd^: Life satisfaction		$\underset {(0.003)}{0.216^{{ ***}}}$	
Cut-point *μ*_1_	$\underset {(0.216)}{-0.474}$	$\underset {(0.219)}{0.899}$	$\underset {(0.215)}{-0.428}$
Cut-point *μ*_2_	$\underset {(0.216)}{0.615}$	$\underset {(0.219)}{2.076}$	$\underset {(0.215)}{0.632}$
Cut-point *μ*_3_	$\underset {(0.216)}{1.746}$	$\underset {(0.219)}{3.261}$	$\underset {(0.215)}{1.728}$
Cut-point *μ*_4_	$\underset {(0.216)}{2.592}$	$\underset {(0.219)}{4.133}$	$\underset {(0.215)}{2.548}$
*ME at mean of absolute income on:*			
Pr(Poor health)	$\underset {(1.92\text {e-}08)}{-2.84\text {e-}07^{{ ***}}}$	$\underset {(1.71\text {e-}08)}{-2.58\text {e-}07^{{ ***}}}$	$\underset {(2.32\text {e-}08)}{-3.02\text {e-}07^{{ ***}}}$
Pr(Fair health)	$\underset {(2.05\text {e-}08)}{-3.06\text {e-}07^{{ ***}}}$	$\underset {(1.95\text {e-}08)}{-2.97\text {e-}07^{{ ***}}}$	$\underset {(2.49\text {e-}08)}{-3.24\text {e-}07^{{ ***}}}$
Pr(Good health)	$\underset {(6.44\text {e-}09)}{8.80\text {e-}08^{{ ***}}}$	$\underset {(4.97\text {e-}09)}{6.65\text {e-}08^{{ ***}}}$	$\underset {(7.80\text {e-}09)}{9.56\text {e-}08^{{ ***}}}$
Pr(Very good health)	$\underset {(1.78\text {e-}08)}{2.65\text {e-}07^{{ ***}}}$	$\underset {(1.68\text {e-}08)}{2.55\text {e-}07^{{ ***}}}$	$\underset {(2.14\text {e-}08)}{2.79\text {e-}07^{{ ***}}}$
Pr(Excellent health)	$\underset {(1.59\text {e-}08)}{2.37\text {e-}07^{{ ***}}}$	$\underset {(1.54\text {e-}08)}{2.34\text {e-}07^{{ ***}}}$	$\underset {(1.92\text {e-}08)}{2.51\text {e-}07^{{ ***}}}$

For AIH, dummies for countries are included but not reported, and available upon request

***: 1% significant; **: 5% significant; *: 10% significant. Standard deviations are in parentheses, below the coefficients.

Coefficients of individual income and income squared provide support for all the hypotheses that there is a positive and concave relationship between income and self-perceived health status. Indeed, coefficients associated to the income variable are all positive and significant and coefficients associated to the income squared variable are all negative and significant. This implies that higher income is related to a better health outcome. As a result, the absolute income hypothesis is verified. Concerning income inequalities, coefficients on the Theil index in columns two and three are negative and significantly different from zero. This supports evidence of the strong version of income inequality hypothesis stating that an increase in income inequalities is detrimental to all members of a society, i.e. income inequalities and health are negatively related. Indeed concerning this index, zero represents an egalitarian state, thus the negative relationship between self-perceived health and the indicator of income inequalities is in line with health being better if the index is low. However, results in column three do not give support to the weak version of income inequality hypothesis which states that inequalities are more detrimental to the least well-off in a society. Indeed, we introduce individual rank (by country) and an interaction term between the rank and the index of income inequalities to allow a variation between income level and the effect of income inequalities. In the specification, we choose to follow the framework of Mellor and Milyo [27] who introduced interaction terms between the measurement of income inequalities and dummies variables based on quintiles of income (1 for the lowest income group and 5 for the highest, which is a proxy for the rank). In other words, interaction terms indicate the effect of aggregate income inequalities (at the country level) on self-perceived health status between individuals situated at different levels of the income distribution. Concerning the first two interaction terms (*II*_*j*_∗*Q*1 and *II*_*j*_∗*Q*2), these indicate the effect of aggregate income inequalities (at the country level) on self-perceived health status between the poorest individuals (situated at the lower end of the income distribution) and the richest ones (reference category corresponding to individuals situated at the top of the income distribution). These coefficients are positive and statistically significant, meaning that for the poorest individuals (compared to more well-off individuals), an increase in income inequalities in their country increases self-perceived health status, which is in contradiction with the weak version of the income inequality hypothesis. Concerning the two other interaction terms (third and fourth quintiles, representing people at the middle and almost top of the income distribution), coefficients are not statistically significant meaning that middle and higher income people are not affected at all by an increase in income inequalities. This claim does not support the weak version because this hypothesis states that people at the lower end are the most affected by an increase in income inequalities compared to people at the top of the income distribution. As a result, higher income people should also be affected by income inequalities (at a lower rate). Our qualitative results suggest that for low-income individuals, an increase in income inequalities in their country is positively related to report a better health status. Furthermore, for higher income individuals, an increase in income inequalities in their country is not related to report neither a better nor a lower health status. To conclude, our results do not support the weak version of income inequality hypothesis, but it further invalidates this weak version because our qualitative results quite claim the opposite.

Regarding the mechanisms of Kawachi and Kennedy [18] (Table 1, column two), the disinvestment in human capital (first mechanism) is characterized by the percentage of health expenditure in the GDP.^9^ The coefficient associated is positively correlated to health meaning that when governments increase health spending, this has a positive effect on individual health. For the second mechanism, we want to illustrate the interaction between individuals to represent the erosion of social capital. As a result, we choose a variable from the SHARE survey: “received help from others”. The coefficient associated to this variable is negative and significant. We can explain this negative association by saying that people who are in bad health are the ones who receive help. In order, to legitimize this explanation, we also do the estimation with the “reverse variable”: “given help to others”. In this case, the coefficient is positive and significant proving that people in good health offer their help. Then, the last mechanism is about social comparisons. The coefficient associated to this variable (“life satisfaction”) is positively linked to health which implies that when individuals are satisfied with their life, they also report having a good health.

In sum, our baseline specifications provide evidence of a statistically significant association between income, income inequalities and health since results are robust to model specifications.

### Robustness checks

As a sake of robustness, we also make our entire analysis using the pooled database (see Table 6 in the Appendix section) and the results are very similar to the ones obtained with the fifth wave of the survey.

**Fig. 4:**
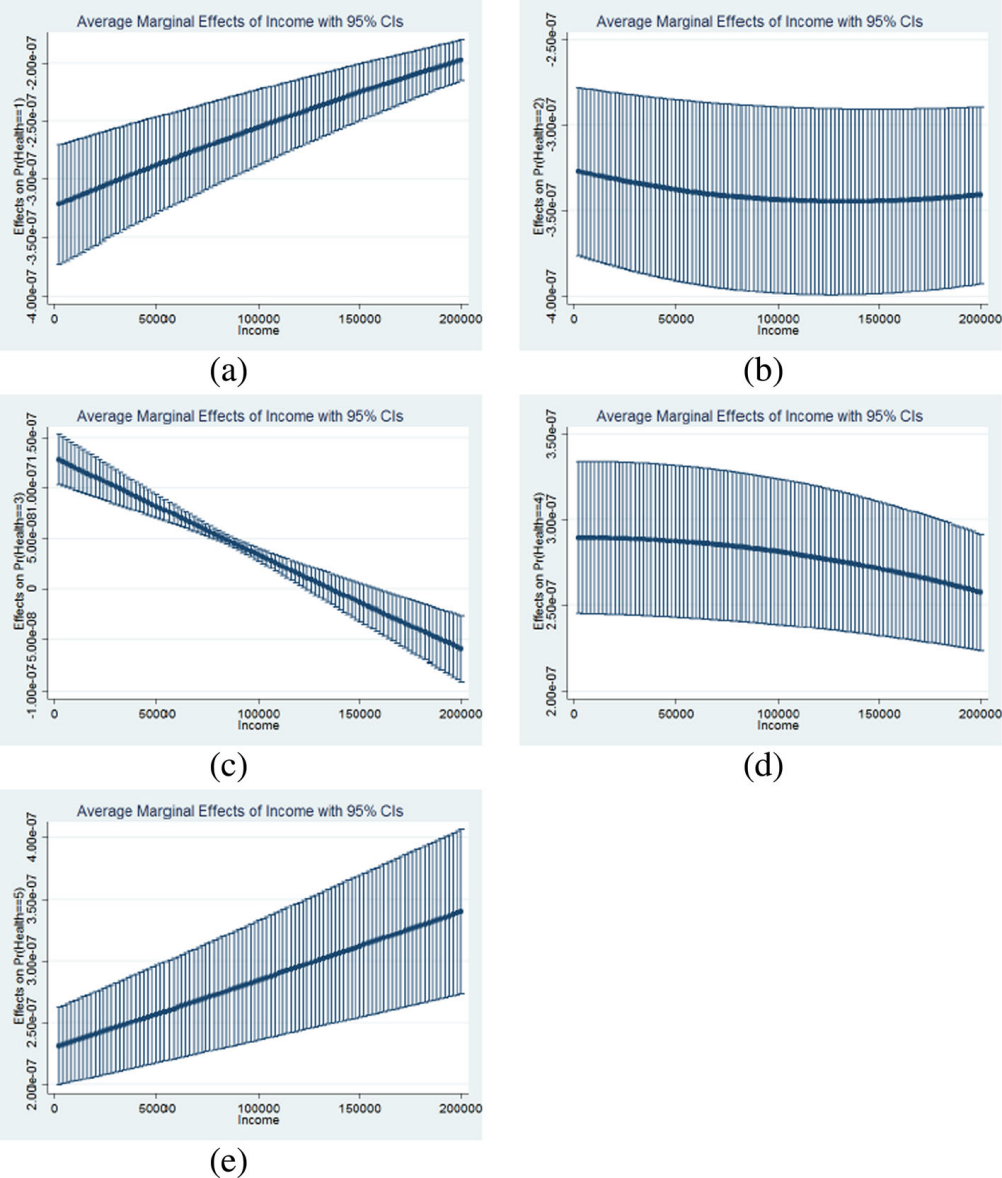
Average marginal effects of income on health - Absolute Income Hypothesis. **a** Probability to report a poor health; **b** Probability to report a fair health; **c** Probability to report a good health; **d** Probability to report a very good health; **e** Probability to report an excellent health

To give more support to the concavity assumption, we compute, for all three hypotheses, the marginal effects at mean^10^ of income on the five outcomes. Results, reported at the end of Table 1, are all significant. On one hand, for the first two outcomes, income has a negative effect on the probability to report either a poor health or a fair health status. On the other hand, there is a positive effect of income on the probability to report being in a good, very good and excellent health (outcomes three to five). These results are obtained following the ordered probit regressions of the three hypotheses, where the quadratic effect of income is investigated (see Eqs. 1, 2 and 3). These results do not validate the concavity assumption but they do show the increasing effect of income on self-perceived health status. We also plot the average marginal effect of income on each outcome for all individuals with a confidence interval, in order to give more support to the concavity effect in the three hypotheses (see Fig. 4). We restrict ourselves to individuals who earn less than 200,000€ per year (which corresponds to more than 99% of the distribution, see Table 4 in the Appendix section for further information on the distribution of income). The following graphs (Fig. 4) concern the absolute income hypothesis.^11^ Graph 4a gives the impact of income on the probability to report a poor health. This impact is negative (y-axis is negative), meaning that when income raises, the probability decreases. In addition, the negative impact is stronger for the majority of the population than for individuals who earn very high incomes. In other words, for low incomes, in absolute terms, an additional increase in income has a larger impact on the probability of reporting a poor health than for very high income. This is a low support for the concavity assumption. Graph 4b gives the impact of income on the probability of reporting a fair health status. Conclusion are similar to the ones of graph 4a since the effect is negative. The slight decreases of the curve at the beginning does not impact the conclusion and can be related to large confidence intervals. Graph 4c gives the impact of income on the probability to report a good health status. For almost all the distribution, when income raises, the probability increases. Then, graphs 4d and 4e are more conclusive. Indeed, graph 4d gives the impact of income on the probability to have a very good health. For more than 99% of the income distribution, this impact is positive and decreasing, which might support the concavity assumption. Finally, graph 4e gives the impact of income on the probability of reporting an excellent health status. As previously, when income increases, the probability to have an excellent health increases. However, when we look at people with very high incomes^12^, this impact is greater than for the majority of individuals.

Finally, it is important to investigate the robustness of our results by taking into account the subjective nature of the self-perceived health status. Indeed, our baseline specification depends on a dependent variable which is subjective. Self-reported measures give a good amount of information about individual health since people summarize all the health information they have from their practitioners (general practitioners and specialists) and from what they feel [1]. The use of this measure in our specification raises the problem of interpersonal comparisons between people aged 50 and over (“Is the way I consider “good health” the same as you consider this health commodity?”. Empirical studies on the relationship between health, income and income inequalities commonly use ordered probit models where the thresholds are constant by assumption. However, one limit is that it restricts the marginal probability effects. In fact the distributional effects are restricted by the specific structure. Then, another limit is that additional individual heterogeneity between individual realizations is not allowed by the distributional assumption. Thus, Boes and Winkelmann [2] and Jones and Schurer [16] both give a solution to these issues with the use of the generalized ordered probit model since it is based on a latent threshold where the thresholds themselves are linear function of the explanatory variables. In other words, previous thresholds of Eq. 8 are now computed by selecting individual characteristics so that they depend on covariates:

9 $$ \mu_{ij} = \widetilde{\mu}_{j} + x_{i}'\gamma_{j}  $$

where *γ*_*j*_ is a vector of response specific parameters. We have:

10 $$  \mu_{ij} = \mu_{j} \qquad \forall_{i}\in C_{j}  $$

where *C*_*j*_ is the class. With this model, the probabilities are:

11 $$ P(y=j|x)=F(\widetilde{\mu}_{j}-x_{i}\beta_{j})-F(\widetilde{\mu}_{j-1}-x_{i}\beta_{j-1})  $$

Now, the effects of covariates on the log-odds are category-specific and this model allows to have more heterogeneity across individuals. Results concerning the generalized ordered probit model are similar to those obtained from the ordered probit model. All the effects are estimated around each four cut-points (from poor to fair, from fair to good, from good to very good, and from very good to excellent). For all the hypotheses (absolute income hypothesis - Appendix: Table 7, income inequality hypothesis, both versions - Tables 8 and 9 in the Appendix part), the coefficients associated to the variables of interest (income and income squared) do not change significantly in comparison to the results with the ordered probit model. Results are consistent (either with the Theil index or the Gini coefficient for the income inequality hypothesis) as this is proved in previous study [22]. In fact, in the four cut-points, the results legitimize the concavity assumption of income since the coefficients are statistically significant. Moreover, the index of income inequalities is negative and significant which is in line with the strong version of the income inequality hypothesis. Then, concerning the interaction terms, these are not significant for all quintile groups which do not justify the weak version of income inequality hypothesis. Finally, adding some heterogeneity in this model and taking into account the issues of interpersonal comparisons do not modify our previous results.

## Conclusion

In this study we underline the hypotheses through which health is associated to income and income inequalities. The aim of this paper is to empirically investigate the evidence for the absolute income hypothesis and both the strong and the weak versions of the income inequality hypothesis for people aged 50 and over in Europe, using data from the SHARE survey. Indeed, we review the relationship on income-related health inequalities where we mention the literature as well as the theoretical and statistical tools needed to carry out this research. Then we present the data used and some descriptive statistics. Finally we show the model specification, the results of the three hypotheses and some robustness tests. This whole work, both the literature study and the establishment of various models led us to estimate different assumptions on the relationship between health and income. This study is one of the first analyzing this relationship through different hypotheses at the same time using the SHARE survey which is a rich database, containing a lot of information on elderly people and countries simultaneously.

We find evidence supporting the absolute income hypothesis which states that people with higher incomes have better health outcomes. We also find evidence supporting the strong version of income inequality hypothesis which argues that inequality affects all members in a society equivalently. In this hypothesis, we find that when there are high income inequalities in a country, people aged 50 and over feel less healthy. However, we do not find evidence supporting the weak version of income inequality hypothesis which states that only the least well-off are hurt by income inequalities in a society. This hypothesis underlines the fact that income inequalities are more detrimental for the health of people with low incomes. Our qualitative results suggest that for low-income individuals, an increase in income inequalities in their country is positively related to report a better health status. Furthermore, for higher income individuals, an increase in income inequalities in their country is not related to report either a better or a lower health status. One limitation is the used of cross-sectional data without investigating possible endogeneity issues. Thus our results highlight statistical associations rather than causal effects. Finally, by implementing the generalized ordered probit, we control for potential problems of interpersonal comparisons and the results are very similar to those found with the ordered probit model.

Results concerning the hypotheses are consistent with the concavity assumption of income on health. Extension would be to highlight causal effects, using other methods, in order to support some political implication. In fact, what is important in determining the health status is more how income is distributed in a society and less the overall health of this society. As a result, the more equally income is distributed, the better the overall health in this society. Concerning political implication, one way to improve health might be to take measures using the redistribution of incomes as a lever. In fact, Lynch et al. [24] argue that, redistributive fiscal and tax policies will help the governments to achieve better population health. Deaton [11] explains that if income inequalities affect health, transfer policies that affect the distribution of incomes would have good effects through individual levels of health. There will be like a virtuous circle in which incomes influence the health status (improving the production possibilities of the economy can be achieved by improving the health) which in turn affects the income.

## Endnotes

^1^ In this way, redistributing income from rich people to poor people will have an important and positive impact on the health of the poorer people, whereas the richer ones will experience a small decrease in their health.

^2^ Such as age, gender, number of years of education, marital status and the job situation. It can also contain countries dummies variables.

^3^ See http://ec.europa.eu/ for an explanation of the European Innovation Partnership on Active and Healthy Ageing - A Europe 2020 initiative.

^4^ After wave four was completed, the average retention rate over the year was 81%.

^5^ For instance, if 50 percent of the population has no income and the other half has the same income, the Gini index is 0.5. The same result can be found with the following analysis which is less unequal. On one hand, 25 percent of total income is shared in the same way by 75 percent of the population, and on he other hand, the remaining 25 percent of the total income is divided by the remaining 25 percent of the population.

^6^ It is this normalized index that we use hereafter and that we name the Theil index.

^7^ Once $h_{i}^{*}$ crosses a certain value you report fair, then poor, then good, then very good, then excellent health.

^8^ Results associated to the Gini coefficient are not provided here but they are very similar and available upon request.

^9^ Source: OECD website.

^10^ We look at the average individual of the database and compute the marginal effects.

^11^ We do not include the ones for the income inequality hypothesis (both versions) since the results are very similar and do not change the main conclusion, but these are available upon request.

^12^ In this case, people with very high incomes are individuals who earn more than 150,000€ per year, corresponding to less than 2% of the sample.

**Table 2:** Descriptive statistics of the variables

Variables	Mean	Standard deviation	Minimum	Maximum
Health				
Self-perceived health status (*N*=63626)	2.85	1.09	1	5
Inequalities				
Gini per country	0.39	0.05	0.31	0.48
Theil per country	0.33	0.19	0.16	0.82
Other Variables				
Income	36,621.21	71,863.78	2	1.00e+07
GDP per country (2013 - Dollar US/capita)	39,726.43	11,543.57	26,160.08	92,781.41
Education	11.12	4.28	1	25
Age	67.12	10.06	50	103

**Table 3:** Detailed descriptive statistics for the health

Health	Percentage of people
Poor (1)	10.81%
Fair (2)	27.01%
Good (3)	36.52%
Very Good (4)	17.58%
Excellent (5)	8.18%

**Table 4:** Detailed descriptive statistics for income

Distribution	Income
5%	3,828.99
25%	12,446
50%	24,659.55
75%	46,200
95%	103,897.2

**Table 5:** Detailed descriptive statistics for the countries

Country	Percentage of people*	GDP - 2013**	Indexes of inequality***	
			Theil index	Gini index
Austria	6.54%	45 132.54	0.1762	0.3222
Germany	8.71%	43 282.31	0.2234	0.3672
Sweden	7.06%	44 585.87	0.1672	0.3183
Netherlands	6.42%	46 749.31	0.2152	0.3543
Spain	9.75%	33 111.45	0.2521	0.3813
Italy	6.88%	34 836.43	0.373	0.4239
France	6.86%	37 617.06	0.8224	0.4772
Denmark	6.37%	43797.23	0.1578	0.3138
Switzerland	4.62%	56 896.91	0.2144	0.3554
Belgium	8.66%	41 863.94	0.3849	0.4545
Czech Republic	8.7%	28 962.64	0.2123	0.3512
Luxembourg	2.5%	92 781.4	0.2649	0.3979
Israel	3.56%	32 504.72	0.2475	0.3906
Slovenia	4.51%	28 675.43	0.3696	0.451
Estonia	8.88%	26 160.08	0.6816	0.4497

*: From each country in the full sample

**: Gross Domestic Product, Total dollar US/capita

***: Values

## Appendix

### Descriptive Statistics

### Additional Econometric Results

**Table 6:** Results of the ordered probit regressions for the pooled database

Variables	Absolute Income	IIH	
	Hypothesis	Strong Version	Weak Version
Income	$\underset {(4.74\text {e-}08)}{1.41\text {e-}06^{{ ***}}}$	$\underset {(4.34\text {e-}08)}{1.94\text {e-}06^{{ ***}}}$	$\underset {(4.76\text {e-}08)}{1.16\text {e-}06^{{ ***}}}$
Income squared	$\underset {(1.14\text {e-}14)}{-1.78\text {e-}13^{{ ***}}}$	$\underset {(1.13\text {e-}14)}{-2.39\text {e-}13^{{ ***}}}$	$\underset {(1.12\text {e-}14)}{-1.46\text {e-}13^{{ ***}}}$
*Quintiles of income: Reference - Q5*			
Quintile 1			$\underset {(0.019)}{-0.379^{{ ***}}}$
Quintile 2			$\underset {(0.019)}{-0.288^{{ ***}}}$
Quintile 3			$\underset {(0.019)}{-0.184^{{ ***}}}$
Quintile 4			$\underset {(0.018)}{-0.115^{{ ***}}}$
Index of inequalities (II) - Theil		$\underset {(0.018)}{-0.473^{{ ***}}}$	$\underset {(0.038)}{-0.567^{{ ***}}}$
Interaction quintile 1 and II			$\underset {(0.053)}{0.121^{{ *}}}$
Interaction quintile 2 and II			$\underset {(0.053)}{0.054}$
Interaction quintile 3 and II			$\underset {(0.052)}{-0.012}$
Interaction quintile 4 and II			$\underset {(0.052)}{0.053}$
Interaction quintile 5 and II			Reference
GDP		$\underset {(3.03\text {e-}07)}{0.0002^{{ ***}}}$	$\underset {(3.06\text {e-}07)}{0.0002^{{ ***}}}$
Age	$\underset {(0.003)}{-0.014^{{ ***}}}$	$\underset {(0.003)}{-0.018^{{ ***}}}$	$\underset {(0.003)}{-0.015^{{ ***}}}$
Age squared	$\underset {(0.00002)}{-0.0001^{{ ***}}}$	$\underset {(0.0002)}{-0.0001^{{ **}}}$	$\underset {(0.00002)}{-0.0006^{{ ***}}}$
Years of education	$\underset {(0.001)}{0.021^{{ ***}}}$	$\underset {(0.0005)}{0.019^{{ ***}}}$	$\underset {(0.001)}{0.017^{{ ***}}}$
Gender =1 if women	$\underset {(0.005)}{-0.055^{{ ***}}}$	$\underset {(0.005)}{-0.057^{{ ***}}}$	$\underset {(0.005)}{-0.050^{{ ***}}}$
*Marital Status: Reference - Married*			
Registered partnership	$\underset {(0.017)}{-0.060^{{ ***}}}$	$\underset {(0.017)}{-0.030^{{ *}}}$	$\underset {(0.017)}{-0.026}$
Married, not living with spouse	$\underset {(0.009)}{-0.098^{{ ***}}}$	$\underset {(0.009)}{-0.087^{{ ***}}}$	$\underset {(0.009)}{-0.091^{{ ***}}}$
Never married	$\underset {(0.014)}{-0.127^{{ ***}}}$	$\underset {(0.013)}{-0.108^{{ ***}}}$	$\underset {(0.014)}{-0.027^{{ **}}}$
Divorced	$\underset {(0.011)}{-0.079^{{ ***}}}$	$\underset {(0.011)}{-0.062^{{ ***}}}$	$\underset {(0.011)}{0.016}$
Widowed	$\underset {(0.009)}{-0.046^{{ ***}}}$	$\underset {(0.009)}{-0.055^{{ ***}}}$	$\underset {(0.009)}{0.026^{{ ***}}}$
*Waves: Reference - Wave 5*			
Wave 1	$\underset {(0.009)}{0.139^{{ ***}}}$	$\underset {(0.009)}{0.431^{{ ***}}}$	$\underset {(0.009)}{0.469^{{ ***}}}$
Wave 2	$\underset {(0.009)}{0.094^{{ ***}}}$	$\underset {(0.009)}{0.247^{{ ***}}}$	$\underset {(0.009)}{0.272^{{ ***}}}$
Wave 4	$\underset {(0.006)}{-0.024^{{ ***}}}$	$\underset {(0.006)}{-0.001}$	$\underset {(0.006)}{0.003}$
Cut-point *μ*_1_	$\underset {(0.104)}{-2.494}$	$\underset {(0.104)}{-1.960}$	$\underset {(0.105)}{-1.976}$
Cut-point *μ*_2_	$\underset {(0.104)}{-1.46}$	$\underset {(0.105)}{-0.952}$	$\underset {(0.105)}{-0.962}$
Cut-point *μ*_3_	$\underset {(0.104)}{-0.378}$	$\underset {(0.104)}{0.106}$	$\underset {(0.105)}{0.102}$
Cut-point *μ*_4_	$\underset {(0.104)}{0.455}$	$\underset {(0.104)}{0.919}$	$\underset {(0.105)}{0.919}$

For AIH, dummies for countries are included but not reported, and available upon request

***: 1% significant; **: 5% significant; *: 10% significant. Standard deviations are in parentheses, below the coefficients

**Table 7:** Absolute Income Hypothesis - Generalized ordered probit (Wave 5)

Variables	Health commodities			
	1 to 2	2 to 3	3 to 4	4 to 5
Income	1.99e-06 ***	2.25e-06 ***	3.68e-06 ***	3.81e-06 ***
	(2.76e-07)	(2.00e-07)	(2.44e-07)	(4.44e-07)
Income squared	-2.11e-13 ***	-7.96e-13 ***	-3.26e-13 ***	-5.41e-12 ***
	(2.90e-14)	(1.17e-13)	(4.71e-13)	(1.55e-12)
Age	0.037 ***	0.037 ***	0.026 ***	0.029 ***
	(0.01)	(0.008)	(0.009)	(0.012)
Age squared	-0.0004 ***	-0.0004 ***	-0.0004 ***	-0.0003 ***
	(0.0001)	(0.0001)	(0.0001)	(0.0001)
Years of education	0.031 ***	0.038 ***	0.036 ***	0.024 ***
	(0.002)	(0.001)	(0.001)	(0.002)
Gender =1 if women	0.066 ***	-0.014	-0.005	-0.002 **
	(0.016)	(0.012)	(0.012)	(0.016)
*Marital Status*:				
Married, living with spouse	Reference group			
Registered partnership	-0.063	-0.093 **	0.029	-0.027
	(0.069)	(0.046)	(0.045)	(0.057)
Married, not living with spouse	-0.251 ***	-0.112 **	-0.0001	0.118 *
	(0.062)	(0.049)	(0.053)	(0.069)
Never married	-0.048	-0.068 ***	-0.038	-0.065 *
	(0.032)	(0.024)	(0.026)	(0.035)
Divorced	-0.157 ***	-0.059 ***	0.05 ***	0.06 **
	(0.026)	(0.019)	(0.021)	(0.027)
Widowed	-0.017	-0.026	0.002	-0.015
	(0.021)	(0.017)	(0.02)	(0.029)
*Job Situation*:				
Retired	Reference group			
Employed	0.398 ***	0.312 ***	0.203 ***	0.174 ***
	(0.029)	(0.019)	(0.019)	(0.025)
Unemployed	-0.222 ***	-0.191 ***	-0.233 ***	-0.126 **
	(0.047)	(0.035)	(0.038)	(0.053)
Permanently sick	-1.196 ***	-1.268 ***	-1.307 ***	-0.963 ***
	(0.033)	(0.038)	(0.054)	(0.076)
Home-maker	-0.088 ***	-0.052 **	-0.047 *	-0.006
	(0.029)	(0.022)	(0.025)	(0.035)
Other	-0.354 ***	-0.173 ***	-0.145 ***	-0.017
	(0.041)	(0.037)	(0.046)	(0.064)

Dummies for countries are included but not reported, and available upon request

***: 1% significant; **: 5% significant; *: 10% significant

1 to 2: Poor to Fair; 2 to 3: Fair to Good; 3 to 4: Good to VG; 4 to 5: VG to Excellent

**Table 8:** IIH, strong version - Generalized ordered probit (Wave 5)

Variables	Health commodities			
	1 to 2	2 to 3	3 to 4	4 to 5
Income	1.75e-06 ***	2.34e-06 ***	3.89e-06 ***	3.20e-06 ***
	(2.69e-07)	(1.97e-07)	(2.38e-07)	(4.42e-07)
Income squared	-1.89e-13 ***	-8.28e-13 ***	-3.75e-12 ***	-5.18e-12 ***
	(2.82e-14)	(1.18e-13)	(4.72e-13)	(1.60e-12)
*Index of inequalities (Theil)*	-0.095 **	-0.369 ***	-0.7389 ***	-0.4746 ***
	(0.041)	(0.031)	(0.035)	(0.048)
*Mechanisms*:				
1st: % Health exp. in the GDP	0.059 ***	0.087 ***	0.073 ***	0.082 ***
	(0.005)	(0.004)	(0.004)	(0.006)
2nd: Received help from others	-0.214 ***	-0.193 ***	-0.134 ***	-0.089 ***
	(0.009)	(0.008)	(0.009)	(0.013)
2nb bis: Given help to others	0.001 ***	0.001 ***	0.001 ***	0.001 ***
	(0.0001)	(0.0001)	(0.0001)	(0.0001)
3rd: Life satisfaction	0.195 ***	0.215 ***	0.239 ***	0.238 ***
	(0.004)	(0.003)	(0.004)	(0.006)
GDP	2.52e-06 ***	1.41e-06 **	-4.87e-07	5.94e-07
	(8.66e-07)	(6.04e-07)	(6.36e-07)	(8.72e-07)
Age	0.019 *	0.004	0.013	0.019 *
	(0.01)	(0.008)	(0.009)	(0.012)
Age squared	-0.0003 ***	-0.0002 ***	-0.0003 ***	-0.0003 ***
	(0.0001)	(0.0001)	(0.0001)	(0.0001)
Years of education	0.025 ***	0.029 ***	0.028 ***	0.021 ***
	(0.002)	(0.001)	(0.0014)	(0.0018)
Gender =1 if women	0.069 ***	-0.018	-0.003	-0.0004
	(0.016)	(0.012)	(0.012)	(0.016)
*Marital Status*:				
Married, living with spouse	Reference group			
Registered partnership	-0.023	-0.053	0.034	0.014
	(0.071)	(0.047)	(0.045)	(0.058)
Married, not living with spouse	-0.131 **	0.005	0.091 *	0.122 *
	(0.065)	(0.051)	(0.054)	(0.072)
Never married	0.033	0.023	0.064 **	0.001
	(0.034)	(0.025)	(0.027)	(0.036)
Divorced	-0.046 *	0.062 ***	0.166 ***	0.122 ***
	(0.028)	(0.021)	(0.022)	(0.028)
Widowed	0.053 **	0.069 ***	0.076 ***	0.022
	(0.023)	(0.018)	(0.022)	(0.031)
*Job Situation*:				
Retired	Reference group			
Employed	0.344 ***	0.225 ***	0.177 ***	0.176 ***
	(0.03)	(0.019)	(0.019)	(0.025)
Unemployed	-0.141 ***	-0.097 ***	-0.11 ***	0.012
	(0.048)	(0.035)	(0.039)	(0.054)
Permanently sick	-1.016 ***	-1.121 ***	-1.098 ***	-0.744 ***
	(0.034)	(0.034)	(0.056)	(0.084)
Home-maker	-0.074 ***	-0.033	-0.076 ***	-0.044
	(0.029)	(0.022)	(0.025)	(0.035)
Other	-0.299 ***	-0.114 ***	-0.09 *	0.048
	(0.043)	(0.038)	(0.048)	(0.067)

***: 1% significant; **: 5% significant; *: 10% significant

1 to 2: Poor to Fair; 2 to 3: Fair to Good; 3 to 4: Good to VG; 4 to 5: VG to Excellent

**Table 9:** IIH, weak version - Generalized ordered probit (Wave 5)

Variables	Health commodities			
	1 to 2	2 to 3	3 to 4	4 to 5
Income	1.97e-06 ***	3.03e-06 ***	5.92e-06 ***	7.65e-06 ***
	(3.06e-07)	(2.43e-07)	(3.15e-07)	(6.10e-07)
Income squared	-2.09e-13 ***	-1.14e-12 ***	-6.03e-12 ***	-1.60e-11 ***
	(3.17e-14)	(1.25e-13)	(5.21e-13)	(1.92e-12)
*Index of inequalities (Theil)*	-0.319 ***	-0.79 ***	-1.077 ***	-0.899 ***
	(0.101)	(0.065)	(0.065)	(0.084)
Quintile 1	-0.145 ***	-0.195 ***	-0.003	0.07
	(0.055)	(0.039)	(0.043)	(0.059)
Quintile 2	-0.099 *	-0.159 ***	-0.014	0.079
	(0.054)	(0.038)	(0.039)	(0.059)
Quintile 3	-0.061	-0.043	0.018	0.025
	(0.054)	(0.037)	(0.037)	(0.047)
Quintile 4	-0.012	-0.02	0.055	0.023
	(0.056)	(0.036)	(0.034)	(0.043)
Quintile 5	Reference group			
Interaction quintile 1 and II	-0.204 *	0.079	-0.039	0.084
	(0.12)	(0.088)	(0.107)	(0.147)
Interaction quintile 2 and II	-0.162	0.097	0.048	0.029
	(0.123)	(0.087)	(0.101)	(0.138)
Interaction quintile 3 and II	-0.163	-0.048	-0.013	0.144
	(0.125)	(0.088)	(0.098)	(0.129)
Interaction quintile 4 and II	-0.058	0.066	0.001	0.098
	(0.132)	(0.088)	(0.093)	(0.124)
Interaction quintile 5 and II	Reference group			
GDP	0.0001 ***	9.96e-06 ***	3.83e-06 ***	2.17e-06 ***
	(8.30e-07)	(6.31e-07)	(6.99e-07)	(9.91e-07)
Age	0.034 ***	0.023 ***	0.029 ***	0.034 **
	(0.01)	(0.008)	(0.008)	(0.011)
Age squared	-0.0004 ***	-0.0003 ***	-0.0004 ***	-0.0004 ***
	(0.0001)	(0.0001)	(0.0001)	(0.0001)
Years of education	0.025 ***	0.029 ***	0.028 ***	0.022 ***
	(0.002)	(0.001)	(0.001)	(0.002)
Gender =1 if women	0.066 ***	-0.016	0.0004	0.007
	(0.015)	(0.011)	(0.012)	(0.016)
*Marital Status*:				
Married, living with spouse	Reference group			
Registered partnership	0.053	0.023	0.075 *	0.049
	(0.067)	(0.045)	(0.044)	(0.056)
Married, not living with spouse	-0.203 ***	-0.091 *	-0.014	0.052
	(0.061)	(0.049)	(0.052)	(0.068)
Never married	0.034	0.014	0.042	-0.008
	(0.033)	(0.024)	(0.026)	(0.035)
Divorced	-0.079 ***	0.009	0.107 ***	0.085 ***
	(0.027)	(0.02)	(0.021)	(0.027)
Widowed	0.024	0.015	0.019	-0.015
	(0.022)	(0.018)	(0.021)	(0.029)
*Job Situation*:				
Retired	Reference group			
Employed	0.374 ***	0.251 ***	0.206 ***	0.188 ***
	(0.029)	(0.019)	(0.018)	(0.024)
Unemployed	-0.188 ***	-0.169 ***	-0.221 ***	-0.128 **
	(0.046)	(0.034)	(0.038)	(0.053)
Permanently sick	-1.162 ***	-1.262 ***	-1.245 ***	-0.923 ***
	(0.032)	(0.033)	(0.054)	(0.08)
Home-maker	-0.062 **	-0.021	-0.081 ***	-0.069 **
	(0.027)	(0.021)	(0.024)	(0.034)
Other	-0.317 ***	-0.152 ***	-0.148 ***	-0.017
	(0.041)	(0.037)	(0.046)	(0.064)

***: 1% significant; **: 5% significant; *: 10% significant

1 to 2: Poor to Fair; 2 to 3: Fair to Good; 3 to 4: Good to VG; 4 to 5: VG to Excellent
